# Divergent roles of circMPP6 and its parental gene MPP6 in non-small cell lung cancer

**DOI:** 10.3389/fcell.2026.1722916

**Published:** 2026-02-03

**Authors:** Weijuan Gao, Feng Pan, Kangping Qiu, Canhui Ou, Fang Tian, Chengtao Yu

**Affiliations:** 1 Affiliated Hospital of Nanjing University of Chinese Medicine, The First Clinical School of Nanjing University of Chinese Medicine, Nanjing, China; 2 Shanghai Chest Hospital, Shanghai Jiao Tong University School of Medicine, Shanghai, China

**Keywords:** circMPP6, circular RNA, lung cancer, MPP6, proliferation, SLC7A11

## Abstract

**Background:**

Circular RNAs (circRNAs) regulate cancer biology, but their relationships with parental genes remain unclear. We characterized circMPP6, derived from MPP6, and its interplay with MPP6 in non-small cell lung cancer (NSCLC).

**Methods:**

circMPP6 was validated by RNase R resistance and Sanger sequencing in A549 cells. Expression of circMPP6 and MPP6 was measured in 10 paired NSCLC tumors and adjacent-tissues. RNA-seq after gain-of-function of circMPP6, MPP6, and SLC7A11 was followed by enrichment analyses and chromosome-level DEG mapping. Proliferation was assessed by CCK-8 and xenografts. Lactate and glutathione were quantified, SLC7A11 protein measured by Western blot, and prognosis analyzed in GEO/TCGA.

**Results:**

MPP6 trended upward in tumors, while circMPP6 was unchanged. circMPP6 and MPP6 were positively correlated in adjacent-tissues but not in tumors. Overexpression of circMPP6 and MPP6 yielded 765 and 334 DEGs, respectively, with shared enrichment of hypoxia-related pathways. 67 genes were upregulated by circMPP6 but downregulated by MPP6, also linked to hypoxia signaling. circMPP6-regulated DEGs were enriched on chromosome 19, whereas MPP6-regulated DEGs clustered on chromosome 17. Functionally, circMPP6 did not alter proliferation, MPP6 enhanced it, and co-expression attenuated MPP6-driven growth *in vitro* and *in vivo*. circMPP6 reduced intracellular lactate and glutathione; MPP6 minimally affected lactate and increased glutathione. Consistently, circMPP6 downregulated SLC7A11, whereas MPP6 upregulated it. High-risk circMPP6-driven signatures and high MPP6 expression associated with poorer prognosis.

**Conclusion:**

circMPP6 and MPP6 exert distinct, partially opposing effects on NSCLC growth. In the context of MPP6 overexpression, circMPP6 counteracts tumor-promoting programs, highlighting functional divergence between circRNAs and their parental genes.

## Introduction

Lung cancer remains the most diagnosed malignancy and the leading cause of cancer-related mortality worldwide. Non-small cell lung cancer (NSCLC) accounts for approximately 85% of cases (Bray et al., 2024). The development and progression of NSCLC are driven in part by dysregulated and mutated protein-coding transcripts in oncogenes and tumor suppressors, exemplified by EGFR mutations, ALK rearrangements, KRAS alterations, and TP53 inactivation (Lim and Ma, 2019). Beyond protein-coding alterations, recent work has highlighted the regulatory roles of circular RNAs (circRNAs) in tumor biology, acting through mechanisms that may be either dependent on or independent of their parental genes.

circRNAs can modulate gene expression as miRNA sponges, protein scaffolds, or templates for translation. For instance, circHIPK3 functions as a miRNA sponge independently of its parental gene HIPK3 (Zheng et al., 2016), whereas circFBXW7 encodes a peptide that augments the tumor-suppressive function of FBXW7 (Yang et al., 2018). In lung cancer, circ-ITCH relieves repression on ITCH mRNA to increase ITCH expression (Wan et al., 2016). Despite these examples, systematic, side-by-side comparisons between circRNAs and their parental protein-coding transcripts from the same locus remain scarce. Such comparisons are essential to disentangle the division of labor—or potential antagonism—between circular and linear transcripts, thereby refining our understanding of gene regulation and informing therapeutic strategy development in NSCLC.

As a class of covalently closed non-coding RNAs, circRNAs have been implicated across diverse tumor types (Zhou et al., 2020). In NSCLC, multiple circRNAs regulate malignant phenotypes via canonical mechanisms. For example, circSATB2 directly binds miR-326 to control FSCN1 expression, promoting proliferation, migration, and invasion (Zhang et al., 2020). CircNDUFB2 forms a TRIM25/circNDUFB2/IGF2BPs ternary complex that facilitates the ubiquitination and degradation of IGF2BPs (Li et al., 2021). Here, we focus on circMPP6 (hsa_circ_0001686), derived from the MPP6 (MAGUK p55 subfamily member 6) gene. Both circMPP6 and its parental gene have received limited investigation, and their biological functions in cancer remain poorly defined. Notably, MPP6 (MAGUK p55 subfamily member 6) should be distinguished from MPHOSPH6 (M-phase phosphoprotein 6), which shares a similar abbreviation but is a distinct gene.

**FIGURE 1:**
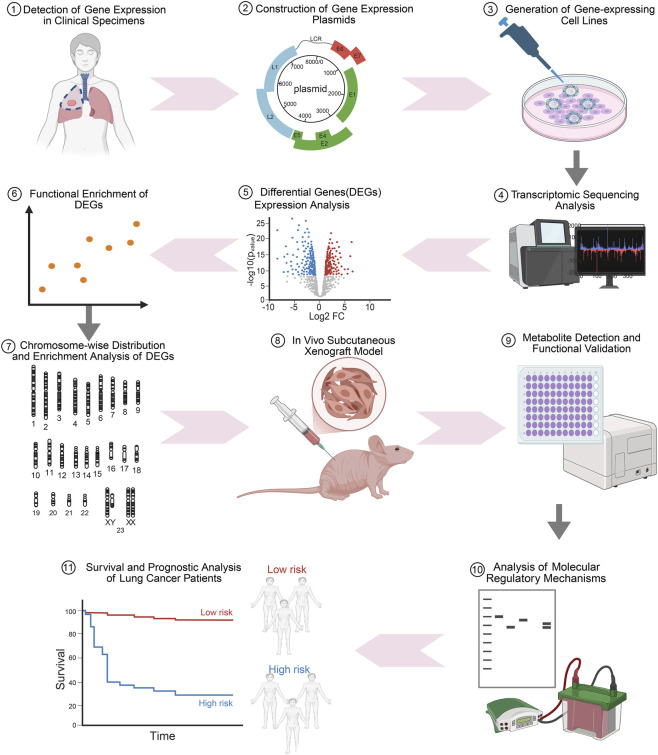
The experimental workflow of study design. Overview of the study, including circMPP6 identification and validation, expression profiling in clinical samples, gain-of-function models (circMPP6, MPP6), RNA-seq and enrichment analyses, chromosome-level mapping, *in vitro* proliferation and metabolic assays, *in vivo* xenograft validation, and prognostic modeling.

In this study, we adopted an integrated approach to delineate the relationship between circMPP6 and its parental gene MPP6 in NSCLC. We first validated circMPP6 in lung cancer cells and profiled the expression of circMPP6 and MPP6 in paired tumor and adjacent tissues from patients with NSCLC. Through gain-of-function models for circMPP6 and MPP6, we performed transcriptome sequencing to identify downstream targets, followed by differential expression and pathway enrichment analyses, chromosomal mapping of differentially expressed genes, and evaluation of glycolysis- and redox-related metabolites and effectors. We further assessed functional outcomes *in vitro* and *in vivo* using A549 proliferation assays and subcutaneous xenograft models, and constructed a prognostic model based on circMPP6-regulated signatures in public NSCLC cohorts. An overview of the study design is provided in Figure 1.

## Materials and methods

### NSCLC clinical sample collection

Ten paired NSCLC tumor and adjacent non-tumor tissues were collected at Shanghai Chest Hospital. Written informed consent was obtained from all participants. The study protocol was approved by the Ethics Committee of Shanghai Chest Hospital (approval ID: LS 1808) and conducted in accordance with the Declaration of Helsinki.

### Cell culture and transfection

A549, H1975 and HEK293T cells were obtained from Zhong Qiao Xin Zhou Biotechnology (Shanghai, China). Cells were cultured in RPMI-1640 (A549, H1975) or DMEM (HEK293T) supplemented with 10% fetal bovine serum (FBS) and 1% penicillin–streptomycin at 37 °C in a humidified incubator with 5% CO2.

Plasmids for overexpression of circMPP6 (hsa_circ_0001686) and MPP6 (NM_016447.4), and for knockdown of SLC7A11 (NM_014331.4), were transfected using TransIntro PL reagent (TransGen Biotech) according to the manufacturer’s instructions. The packaging of lentivirus was co transfected with psPAX2 and pMD2G. Transfection efficiency was assessed by qRT-PCR and/or Western blotting as appropriate.

### RNA extraction and PCR analysis

Total RNA was extracted using TRIzol reagent (TransGen Biotech) following the manufacturer’s protocol. For circRNA validation, total RNA was treated with RNase R (New England Biolabs) to remove linear RNAs.

First-strand cDNA synthesis was performed using HiScript III First Strand cDNA Synthesis SuperMix (YEASEN). PCR products were purified and resolved on 1% agarose gels. The back-splice junction of circMPP6 was confirmed by Sanger sequencing.

### Real-time quantitative PCR (qRT-PCR)

qRT-PCR was performed using Hieff UNICON® qPCR SYBR Green Master Mix (YEASEN). Relative expression was calculated by the 2^(-ΔΔCT)^ method with Actin as the reference gene. The following primers were used: circMPP6:F-GCTCAAGGTGTAGGCCGAAG; R-GGGCAGCTCCGTAAGGTTTT. Actin: F- CTC​ACC​ATG​GAT​GAT​GAT​ATC​GC; R- GGA​ATC​CTT​CTG​ACC​CAT​GCC. MPP6: F- GGA​GGC​CCA​TGA​TAT​TGT​GGC; R- TTC​AAC​CCT​AAA​TGT​CAC​ACC​C. NDRG1: F- CTC​CTG​CAA​GAG​TTT​GAT​GTC​C; R- TCA​TGC​CGA​TGT​CAT​GGT​AGG. DDIT4: F- TGA​GGA​TGA​ACA​CTT​GTG​TGC; R- CCA​ACT​GGC​TAG​GCA​TCA​GC. STC2: F-GCGTGCAGGTTCAGTGTGA; R- GGC​CAG​TCT​CCC​TAC​TGC​T. ANGPTL4: F- GGC​TCA​GTG​GAC​TTC​AAC​CG; R- CCG​TGA​TGC​TAT​GCA​CCT​TCT.

### RNA sequencing and analysis

RNA sequencing of A549 cells with circMPP6 overexpression, MPP6 overexpression, or SLC7A11 knockdown, along with corresponding controls, was performed by Tsingke (Beijing, China). Library construction used poly(A)+ selection or rRNA depletion as specified by the vendor.

Differential expression analysis applied thresholds of |log2 fold change| ≥ 0.58 and adjusted *P* ≤ 0.05. Pathway enrichment was conducted using MSigDB Hallmark and KEGG gene sets. Pathway intersections were visualized with UpSet plots.

### Western blot analysis

Primary antibodies: Vinculin (Proteintech, 66305-1-Ig), MPP6 (Proteintech, 11575-1-AP), and SLC7A11 (Abcam, ab307601). HRP-conjugated secondary antibodies: goat anti-mouse IgG (TransGen Biotech, HS201-1) and goat anti-rabbit IgG (TransGen Biotech, HS101-1).

Proteins were extracted in RIPA buffer containing protease inhibitors and quantified by BCA assay (YEASEN). Equal amounts of protein were resolved by SDS-PAGE, transferred to PVDF membranes, probed with the indicated antibodies, and detected by enhanced chemiluminescence (Bio-Rad). Band intensities were quantified using ImageJ.

### L-lactate and glutathione assay

Intracellular L-lactate and total glutathione were quantified using Beyotime L-Lactate Assay Kit (WST-8 method) and Beyotime Total Glutathione Assay Kit, respectively, following the manufacturers’ protocols. Standard curves were established for concentration calculations, and absorbance was measured on a microplate reader.

### CCK-8 assays

A549 and H1975 cells under the indicated treatments were seeded in 96-well plates at 5 × 10^3^ cells/well and incubated for 24, 48, and 72 h. After adding 10 μL CCK-8 reagent (Cowin Biosciences) per well, plates were incubated at 37 °C for 1 h. Absorbance at 450 nm was measured using a microplate reader. Each condition included at least three technical replicates and was repeated in independent experiments.

### Nude mouse xenograft experiments

All animal procedures were approved by the Animal Ethics Committee of Nanjing University of Chinese Medicine (approval ID: 202501A038) and complied with institutional guidelines for laboratory animal welfare. Male BALB/c nude mice (4–6 weeks old; GemPharmatech) were maintained under specific pathogen-free conditions with a 12 h light/dark cycle and *ad libitum* access to food and water. A549 cells (Control, MPP6, or MPP6 + circMPP6) in logarithmic growth phase were washed with PBS and resuspended in serum-free medium at 5 × 10^7^ cells/mL. A total of 5 × 10^6^ cells in 100 μL were injected subcutaneously into the right flank of each mouse. Tumor length and width were measured with calipers every 2–3 days. Tumor volume was calculated as (length × width^2^)/2. At endpoint, mice were euthanized and tumors were excised and weighed.

### Public data acquisition and analysis

Public datasets were obtained from The Cancer Genome Atlas (TCGA), UCSC Xena (https://xenabrowser.net/datapages/), and the Gene Expression Omnibus (GEO). Specifically, TCGA-LUAD data were accessed via UCSC Xena (Goldman et al., 2020) GEO cohorts GSE72094 and GSE68465 were downloaded with accompanying clinical and survival information (Barrett et al., 2013). The prognostic analysis of MPP6 was performed using the GEPIA web server (http://gepia.cancer-pku.cn/). The RNA and protein expression levels of MPP6 were analyzed using the TCGA and CPTAC datasets.

### R software and packages for bioinformatic analysis

R software (version 4.0.3) was used for data visualization. The volcano plots, heatmaps, trend analysis plots, forest plots, and survival analysis plots in this study were all generated using this software. Key packages such as tidyverse, ggplot2, pheatmap, survival, survminer were utilized. The source code for the analysis plots has been included in the raw data files and uploaded to the database for preservation.

### Statistical analysis

Data obtained were expressed as mean ± standard deviation (SD) and compared between two groups using Student’s t-test and multiple comparisons using ANOVA. *P* < 0.05 was considered statistically significant (*P < 0.05, **P < 0.01, ***P < 0.001). Experiments were performed at least triplicate. Statistical analysis was performed using R software (version 4.0.3) and GraphPad Prism 9.5.

## Results

### Characterization and expression patterns of MPP6 and circMPP6 in NSCLC

**FIGURE 2:**
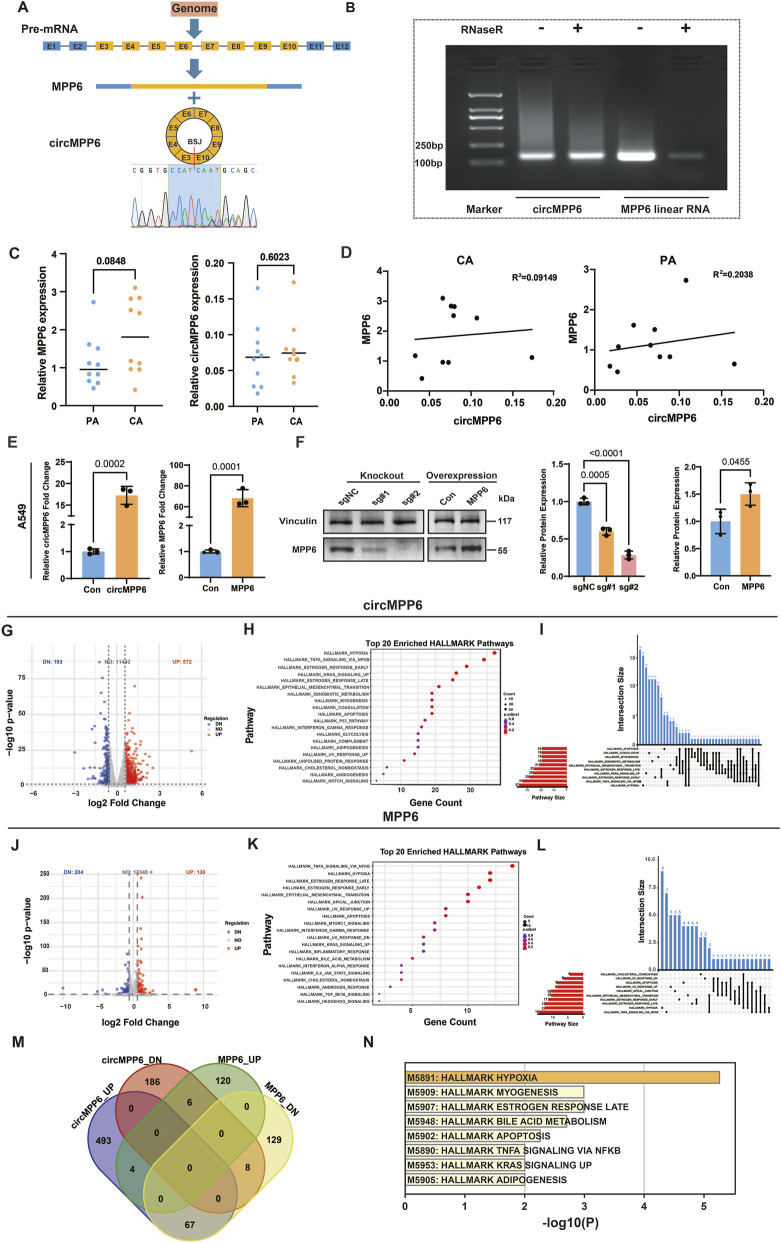
Characterization and expression analysis of MPP6 and circMPP6 in NSCLC. **(A)** Schematic of exon composition for MPP6 mRNA and circMPP6 (hsa_circ_0001686), showing the back-splice junction between exon 10 and exon 3. **(B)** RNase R treatment demonstrating resistance of circMPP6 and depletion of linear MPP6 mRNA relative to mock control. **(C)** qRT-PCR analysis of circMPP6 and MPP6 expression in paired NSCLC tumor (CA) and adjacent non-tumor (PA) tissues (n = 10 pairs). Bars show mean ± SD; statistical tests as indicated. **(D)** Correlation between circMPP6 and MPP6 expression in PA and CA tissues. **(E)** qRT-PCR confirming overexpression of circMPP6 and MPP6 in A549 cells. **(F)** Western blot validating MPP6 overexpression and CRISPR-mediated MPP6 knockout in A549; Vinculin as loading control. **(G,J)** Volcano plots of DEGs upon overexpression of circMPP6 **(G)** or MPP6 **(J)** in A549 cells. Red and blue denote upregulated and downregulated genes, respectively (|log2FC| ≥ 0.58; adjusted P ≤ 0.05). **(H,K)** Pathway enrichment (Hallmark) for DEGs regulated by circMPP6 **(H)** and MPP6 **(K)**; bubble size reflects gene ratio and color reflects FDR. **(I,L)** UpSet plots showing gene overlaps among significantly enriched pathways after circMPP6 **(I)** and MPP6 **(L)** overexpression. **(M)** Venn diagram illustrating DEG overlaps between circMPP6 and MPP6 conditions, highlighting genes with opposite regulation. **(N)** Enrichment analysis of the 67 genes upregulated by circMPP6 but downregulated by MPP6 (Metascape); top pathways are shown.

To verify that circMPP6 is a circular RNA and is present in A549 cells, we designed divergent primers spanning the predicted back-splice junction. By using Sanger sequencing, the back-splice junction sequence “CCAT-CAAT” was confirmed (Figure 2A). Consistently, RNase R digestion assay showed that circMPP6 levels were retained relative to mock treatment, whereas the linear MPP6 mRNA was markedly reduced (Figure 2B). Above results suggested that circMPP6 is circular RNA and is present in A549 cells. In a cohort of 10 paired NSCLC specimens, qRT-PCR detected no significant overall differences in circMPP6 or MPP6 expression between cancer (CA) and paired adjacent (PA) tissues (both P > 0.05), although MPP6 showed an upward trend in CA (*P* = 0.084) (Figure 2C). In TCGA database, MPP6 mRNA level is significantly upregulated in both LUSC and LUAD using a threshold of |log2FC| > 0.5 and P < 0.01. However, when applying a more stringent cutoff (|log2FC| > 1, P < 0.01), only LUSC retains significant upregulation while LUAD shows an upward trend without reaching significance. In CPTAC database, MPP6 protein level is upregulated in LUSC but shows no significant change in LUAD (Supplementary Figure S1). Interestingly, in our PA samples, MPP6 and circMPP6 expression levels were positively correlated, whereas this correlation was disrupted in CA samples (Figure 2D).

### Transcriptomic analysis of gene expression pattern after circMPP6 and MPP6 overexpression

To investigate the downstream biological effects of circMPP6 and MPP6, we established cell lines with overexpression of each in both A549 and H1975 cells, qRT-PCR confirmed overexpression for both circMPP6 and MPP6 (Figure 2E; Supplementary Figure S2A). Western blotting further demonstrated a marked increase in MPP6 protein in the overexpression cells. Conversely, in CRISPR-mediated MPP6 knockout cells, the MPP6 protein band at the expected molecular weight was markedly reduced, supporting the specificity of the detected signal and the correctness of the expression modulation (Figure 2F).

Next, we performed RNA-seq analysis to investigate the effects of circMPP6 and MPP6 overexpression on global gene expression profiles in A549. Upon circMPP6 overexpression, a total of 765 differentially expressed genes (DEGs) were identified (|log_2_FC| ≥ 0.58, adjusted *P*-value ≤0.05), including 572 upregulated and 193 downregulated genes (Figure 2G; Tables 1, 2). Functional enrichment analysis using the Hallmark gene set collection revealed significant enrichment of pathways such as hypoxia and TNFA signaling (Figure 2H). UpSet plot analysis of gene overlap among enriched pathways demonstrated that the hypoxia and TNFA signaling pathways shared relatively few genes with other pathways, indicating that these enrichments are relatively independent and not driven by broadly shared genes (Figure 2I). Similarly, MPP6 overexpression resulted in 334 DEGs, with 130 genes upregulated and 204 genes downregulated (Figure 2J; Table 3, 4). Enrichment analysis again highlighted hypoxia and TNFA signaling as significantly affected pathways (Figure 2K). The UpSet plot further showed that genes involved in hypoxia and TNFA pathways also had limited overlap with other pathways, supporting the specificity of these enrichments (Figure 2L).

**TABLE 1:** Top10 upregulated genes after circMPP6 over-expression screened by fold change and P-value.

Gene_name	Log_2_FC	P value	pAdj
CA9	5.294601	2.39E-27	3.85E-25
CCDC33	2.553526	5.98E-15	2.37E-13
SLPI	2.305968	2.16E-23	2.34E-21
MUC16	2.167933	2.76E-17	1.43E-15
LOX	2.152751	3.97E-11	8.73E-10
SEPP1	2.100951	1.55E-07	1.68E-06
CP	2.024733	2.84E-25	3.90E-23
FAM83A	1.866505	5.19E-08	6.27E-07
ALDOC	1.85876	2.64E-20	2.07E-18
GPNMB	1.852029	2.07E-08	2.71E-07

**TABLE 2:** Top10 downregulated genes after circMPP6 over-expression screened by fold change and P-value.

Gene_name	Log_2_FC	P value	pAdj
CEACAM5	−2.96907	1.89E-26	2.81E-24
CPA4	−2.34076	1.30E-14	4.84E-13
ANXA8	−2.02618	1.39E-06	1.21E-05
ANKRD1	−1.883	3.31E-15	1.34E-13
CYP1A1	−1.76781	5.69E-07	5.39E-06
CEACAM6	−1.74334	2.09E-43	1.60E-40
GRIN2B	−1.66283	9.41E-09	1.33E-07
EDN2	−1.58678	2.85E-07	2.89E-06
MARCH4	−1.56716	3.40E-15	1.37E-13
FOSL1	−1.5563	9.93E-46	1.10E-42

**TABLE 3:** Top10 upregulated genes after MPP6 over-expression screened by fold change and P-value.

Gene_name	logFC	P value	pAdj
PALS2	2.550712	0	0
NID2	1.387261	4.31E-44	1.97E-41
ID4	1.352537	1.09E-22	1.98E-20
NEDD4	1.291396	6.64E-207	3.03E-203
GFRA1	1.288299	3.01E-32	9.35E-30
TENM3	1.216433	4.80E-17	4.75E-15
GIT1	1.172072	4.33E-120	1.18E-116
FZD6	1.149638	1.08E-70	1.23E-67
SCN9A	1.13466	7.13E-23	1.32E-20
PRC1	1.13078	2.41E-99	4.70E-96

**TABLE 4:** Top10 downregulated genes after MPP6 over-expression screened by fold change and P-value.

Gene_name	Log_2_FC	P value	pAdj
MSTRG.19014.22	−10.0002	9.02E-17	8.53E-15
IGF2	−1.67721	7.20E-17	7.03E-15
STC1	−1.13568	4.33E-26	9.72E-24
TFPI	−1.12971	4.52E-66	4.12E-63
SLC40A1	−0.88714	5.97E-20	8.16E-18
TP53INP1	−0.87831	7.07E-25	1.51E-22
AGR2	−0.85888	1.03E-88	1.75E-85
KRT19	−0.77581	6.18E-58	4.70E-55
SMOC1	−0.76428	3.32E-17	3.32E-15
NDRG4	−0.72594	1.37E-15	1.11E-13

To further compare the effects of circMPP6 and MPP6, we used a Venn diagram to assess the overlap of DEGs. Interestingly, 67 genes were found to be upregulated by circMPP6 but downregulated by MPP6 (Figure 2M). Functional analysis of these 67 genes revealed continued enrichment in hypoxia pathways (Figure 2N). Additionally, we performed experiments to validate hypoxia-related transcriptional changes in NSCLC cells with altered circMPP6 or MPP6 expression. Based on a hypoxia-related gene set, we identified four representative hypoxia-associated genes (DDIT4, STC2, ANGPTL4, and NDRG1). In both A549 and H1975 cells, qRT-PCR analysis showed that circMPP6 overexpression significantly upregulated the expression of all four genes, whereas MPP6 overexpression led to a consistent downregulation of these genes (Supplementary Figure S3). These results are in good agreement with our enrichment analysis and support the notion that circMPP6 and MPP6 exert opposing effects on hypoxia-related gene expression programs. Collectively, these findings indicate that both circMPP6 and MPP6 modulate similar key pathways may related to hypoxia signaling, but they can regulate same sets of genes in opposite directions.

### Chromosome-level analysis reveals distinct patterns of circMPP6 and MPP6 regulated gene

**FIGURE 3:**
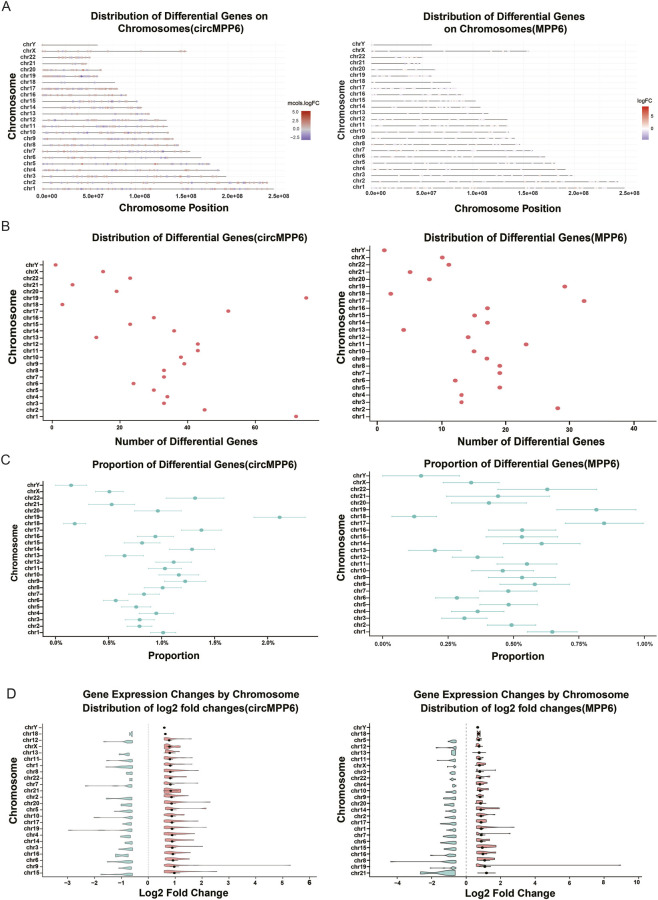
Chromosome-level analysis reveals distinct patterns of circMPP6-regulated and MPP6-regulated gene expression. **(A)** Genome-wide mapping of DEGs to chromosomal coordinates for circMPP6 and MPP6 conditions; red and blue denote up and downregulated genes. **(B)** Counts of DEGs per chromosome for circMPP6 and MPP6. **(C)** Relative enrichment of DEGs per chromosome normalized to total annotated genes on each chromosome. **(D)** Violin plots of absolute fold-change distributions across chromosomes; black dots indicate mean or median values as specified. Chromosomes are ordered by mean absolute fold change.

To compare chromosome-level regulatory patterns of circMPP6 and MPP6, we analyzed their respective transcriptional signatures in parallel. For the chromosomal distribution of DEGs, circMPP6 exhibited the highest burden on chromosome 19 (>60 genes) and the lowest on the Y chromosome (<5 genes), whereas MPP6 overexpression yielded the greatest DEG count on chromosome 17 (>30 genes), with chromosome 19 ranking second (∼30 genes) (Figures 3A,B). SAfter normalizing by total gene content, circMPP6 remained most enriched on chromosome 19 (>2%), while MPP6 showed the higher proportional burdens on chromosomes 17 and 19 (each >0.75%) (Figure 3C). Regarding the magnitude of expression changes, circMPP6 elicited the largest average fold change on chromosome 15, with chromosome 19 ranking approximately eighth; in contrast, MPP6 produced the strongest average fold change on chromosome 21, with chromosome 19 ranking second (Figure 3D). Chromosome-specific pathway enrichment indicated that circMPP6-regulated DEGs on chromosomes 1 and 3 were significantly enriched for hypoxia response and TNFA signaling, consistent with genome-wide patterns, whereas MPP6-regulated DEGs demonstrated significant enrichment of TNFA signaling restricted to chromosome 9 and showed no significant enrichment of hypoxia-related pathways on any chromosome (Supplementary Figures S4, S5). Collectively, these comparative analyses indicate that circMPP6 confers a broader chromosomal footprint with concurrent enrichment of hypoxia and TNFA signaling, particularly on chromosomes 1 and 3, while MPP6 displays a more restricted distribution with a predominantly TNFA-focused signature and distinct chromosomal focal points.

### circMPP6 and MPP6 differentially regulate proliferation and downstream targets

**FIGURE 4:**
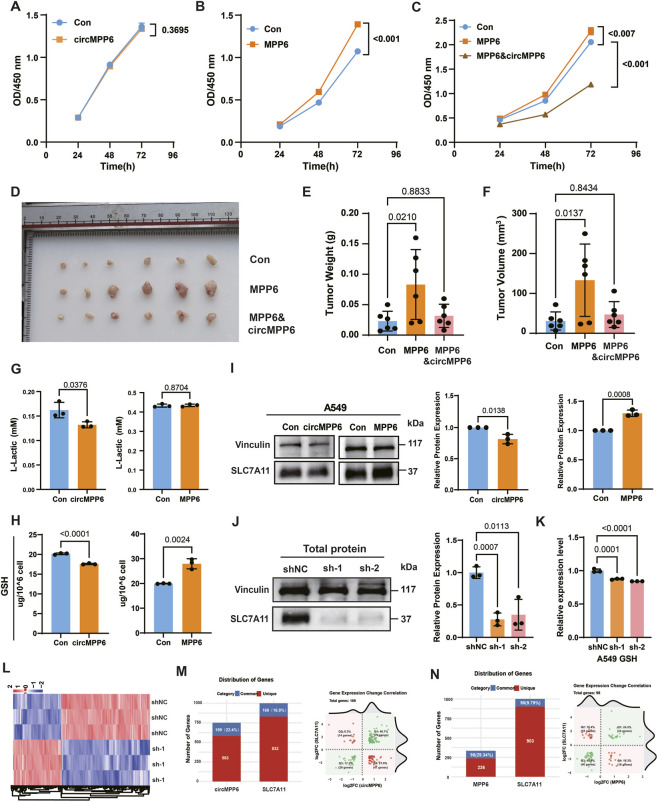
circMPP6 and MPP6 differentially regulate proliferation and metabolic process. **(A–C)** CCK-8 assays evaluating effects of circMPP6 overexpression **(A)**, MPP6 overexpression **(B)**, and co-expression **(C)** on A549 proliferation at indicated time points. **(D–F)** Nude mouse subcutaneous xenografts showing tumor growth under Control, MPP6, and MPP6 and circMPP6 conditions: **(D)** representative tumors, **(E)** tumor weight, **(F)** tumor volume. **(G,H)** Intracellular L-lactate **(G)** and total glutathione (GSH) **(H)** levels in A549 cells following circMPP6 or MPP6 overexpression; normalized as indicated. **(I)** Western blot of SLC7A11 protein in the indicated groups; Vinculin as loading control. **(J)** Western blot confirming SLC7A11 knockdown in A549 cells. **(K)** Total GSH levels following SLC7A11 knockdown. **(L)** Hierarchical clustering heatmap of DEGs after SLC7A11 knockdown. **(M)** Overlap between circMPP6-regulated DEGs and SLC7A11-regulated DEGs: bar plot showing overlap counts; inset pie chart shows concordant vs. discordant regulation. **(N)** Overlap between MPP6-regulated DEGs and SLC7A11-regulated DEGs: bar plot showing overlap counts; inset pie chart shows concordant vs. discordant regulation.

We next evaluated the effects of circMPP6 and MPP6 on A549 and H1975 cell proliferation and related downstream targets. CCK-8 assays demonstrated that circMPP6 overexpression did not significantly affect proliferation (Figure 4A; Supplementary Figure S2C), whereas MPP6 overexpression markedly enhanced proliferative capacity (Figure 4B; Supplementary Figure S2B). Notably, co-expression of circMPP6 with MPP6 substantially attenuated the pro-proliferative effect of MPP6 (Figure 4C; Supplementary Figure S2D). The results obtained in H1975 cells were consistent with those in A549 cells. Consistent with these *in vitro* findings, subcutaneous xenograft experiments in nude mice showed that MPP6 promoted tumor growth, while co-expression of circMPP6 and MPP6 significantly mitigated MPP6-driven tumor expansion (Figures 4D–F).

Given the reported link between hypoxia and glycolysis (Khan et al., 2018; Lee et al., 2020). We employed CoCl_2_ treatment to establish a cellular hypoxia model. The results demonstrated that under CoCl_2_ treatment, overexpression of circMPP6 promoted cell viability in A549 cells (Supplementary Figure S6B), whereas no such effect was observed in H1975 cells (Supplementary Figure S6D). In contrast, MPP6 enhanced cell viability in both A549 and H1975 cell lines (Supplementary Figures S6A, C). These findings suggest that, at least in A549 cells, activation of the hypoxia pathway may be a prerequisite for circMPP6 to exert its pro-viability function. Conversely, the ability of MPP6 to promote cell viability appears to be independent of HIF1 pathway activation.

Additionally, we profiled glycolysis-associated metabolites. circMPP6 overexpression significantly reduced intracellular L-lactate levels, whereas MPP6 overexpression had no significant effect (Figure 4G). Furthermore, circMPP6 led to a pronounced decrease in intracellular glutathione (GSH), while MPP6 increased GSH levels (Figure 4H). Because SLC7A11 is a key determinant of cystine uptake and GSH biosynthesis, we examined its expression. Western blotting revealed that circMPP6 reduced SLC7A11 protein levels, whereas MPP6 increased SLC7A11 (Figure 4I). Similarly, we observed consistent results in H1975 cells (Supplementary Figures S2E, F). In line with its canonical role, SLC7A11 knockdown decreased intracellular GSH (Figures 4J,K). Collectively, these results indicate that circMPP6 and MPP6 exert distinct-and in some contexts opposing-effects on cell proliferation and metabolism, with circMPP6 may suppress GSH levels via downregulation of SLC7A11, and MPP6 may enhance proliferation concomitant with upregulation of SLC7A11.

Previous evidence indicated that circMPP6 and MPP6 could oppositely regulate the expression of SLC7A11. We next sought to investigate whether SLC7A11 serves as one of their downstream effector targets through transcriptomic analysis. Subsequently, RNA sequencing analysis of the SLC7A11 knockdown cells revealed differentially expressed genes (Figure 4L; Tables 5, 6). Notably, 169 genes overlapped with those regulated by circMPP6 (approximately 16.9%), with approximately 63.9% of these genes showing concordant regulatory directions (Figure 4M). Similarly, we compared the downstream differential genes of MPP6 and SLC7A11. The results showed that 29.34% of MPP6-regulated differential genes overlapped with those altered after SLC7A11 knockdown. Among these, 34.7% were regulated in the opposite direction as MPP 6 (Figure 4N).

**TABLE 5:** Top10 upregulated genes after SLC7A11 knockdown screened by fold change and P-value.

Gene_name	log_2_FC	P value	FDR
MSTRG.138	11.0269	1.73E-20	1.65E-19
OR51E1	8.082924	1.21E-10	6.23E-10
VIL1	6.705615	4.57E-06	1.49E-05
FGB	5.093179	5.19E-255	1.16E-252
FXYD2	4.94673	2.24E-157	2.27E-155
ENSG00000268173	4.925985	4.46E-08	1.84E-07
MSTRG.3497	4.473638	0	0
FXYD6	4.364999	4.38E-16	3.37E-15
SCEL	4.050624	4.57E-130	3.42E-128
HAND1	3.969773	4.67E-30	6.58E-29

**TABLE 6:** Top10 downregulated genes after SLC7A11 knockdown screened by fold change and P-value.

Gene_name	log2FC	P value	FDR
SPECC1L-ADORA2A	−8.73183	1.93E-12	1.16E-11
MSTRG.10181	−4.54213	5.19E-86	2.35E-84
HHIP	−3.8632	5.16E-22	5.27E-21
SPOCK1	−3.80339	1.03E-93	5.15E-92
TSPAN18	−3.57972	1.03E-141	8.82E-140
CRISPLD2	−3.56248	4.80E-30	6.75E-29
MYPN	−3.53609	7.49E-35	1.24E-33
ITGB3	−3.41401	9.82E-52	2.51E-50
FOXS1	−3.19587	3.49E-12	2.05E-11
RUNX2	−3.14268	9.51E-18	7.91E-17

### Construction and validation of a circMPP6-regulated gene-based prognostic model for LUAD patients

Lastly, we investigated whether the genes regulated by circMPP6 could be utilized to construct a prognostic model for lung adenocarcinoma (LUAD) patients. Initially, we developed a prognostic diagnostic model based on data from TCGA-LUAD patients, with a forest plot illustrating the prognostic factors included in the model (Supplementary Figures S7A, B). We then validated this model using two independent datasets, GSE72094 and GSE68465. The results demonstrated that both datasets exhibited a trend where the low-risk group had better prognosis, and the high-risk group had poorer prognosis. However, statistical significance was achieved only in the GSE72094 dataset, whereas the model did not reach significance in the GSE68465 dataset (Supplementary Figures S7C, D). We also analyzed the impact of MPP6 expression on prognosis using TCGA data. We found that high MPP6 expression was significantly associated with reduced overall survival and disease-free survival in LUAD patients, suggesting that MPP6 serves as a high-risk prognostic factor for LUAD (Supplementary Figures S7E, F).

## Discussion

In this study, we delineate the relationship between circMPP6 and its parental gene MPP6 in NSCLC and show that they share pathway-level convergence yet display distinct, and at times opposing, functional outputs. Structurally, circMPP6 comprises eight exons generated by back-splicing between exon 10 and exon 3, whereas the linear MPP6 transcript contains twelve exons. Despite the longer sequence of MPP6, gain-of-function experiments indicated that both circMPP6 and MPP6 intersect with hypoxia-related programs. This observation raises the possibility that functional elements within exons 3-10 contribute to pathway engagement; however, this inference remains tentative without exon-centric perturbations.

In clinical samples, circMPP6 expression did not differ significantly between tumors and adjacent tissues, whereas MPP6 showed an upward trend in tumors. Although the *P* value exceeded 0.05, the directionality may reflect limited power given the small cohort. Notably, circMPP6 and MPP6 were positively correlated in adjacent tissues but decoupled in tumors. Together with functional assays showing that circMPP6 attenuates MPP6-driven proliferation, these findings support a model in which coordinated circ-linear expression is maintained in normal tissues to temper growth signals, whereas tumorigenesis disrupts this balance, enabling MPP6-dominant pro-growth activity. Whether such antagonistic coordination is a broader feature of circRNAs and their parental genes and how this impacts tissue homeostasis merit systematic investigation.

Given the shared enrichment in hypoxia pathways, we examined glycolysis- and redox-linked metabolites. circMPP6 overexpression reduced intracellular lactate and GSH, consistent with a shift in metabolic state, whereas MPP6 increased GSH with minimal effect on lactate. Mechanistically, SLC7A11-a key cystine transporter that fuels GSH biosynthesis—was downregulated by circMPP6 and upregulated by MPP6. Transcriptomic comparisons further revealed substantial overlaps between circMPP6- or MPP6-regulated genes and those altered upon SLC7A11 knockdown, with differing degrees of concordance, suggesting that SLC7A11 contributes to, but does not fully explain, their transcriptomic programs.

SLC7A11 is central to redox homeostasis and has been implicated in tumor metabolic reprogramming, glycolysis, and ferroptosis susceptibility (Koppula et al., 2018). Prior studies identified transcriptional regulators of SLC7A11 (e.g., TP53, NRF2, ATF4) that indirectly influence ferroptosis via modulation of cystine import and GSH synthesis (Jiang et al., 2015; Wang et al., 2022; He et al., 2023). By contrast, evidence for circRNA-mediated regulation of SLC7A11 remains limited, with reports of ceRNA mechanisms (e.g., circTTC13, circRPPH1) modulating SLC7A11 expression (Liu et al., 2023; Zhang et al., 2025). We also explored potential mechanisms by which circMPP6 regulates SLC7A11. Using online databases, we predicted miRNAs that may bind circMPP6. CircAtlas (miRanda/TargetScan) identified 24 candidate miRNAs, and CircInteractome predicted 40; intersection analysis yielded five overlapping miRNAs (hsa-miR-623, hsa-miR-649, hsa-miR-936, hsa-miR-1305, and hsa-miR-188-3p). We also screened putative circMPP6-interacting RBPs and identified several candidates, including AGO2 and IGF2BP1 (Supplementary Figure S8). However, to our knowledge, there is currently no direct evidence linking these RBPs to SLC7A11 regulation, making an RBP-based model less compelling at this stage.

We therefore focused on a miRNA-mediated mechanism. Bioinformatic analysis of the SLC7A11 3′UTR revealed putative binding sites for hsa-miR-649, hsa-miR-936, hsa-miR-1305, and hsa-miR-188-3p, with higher predicted scores for hsa-miR-188-3p and hsa-miR-1305. RNA pull-down assays further supported binding of circMPP6 to hsa-miR-188-3p and hsa-miR-1305 (Supplementary Figure S9).

Notably, our data do not fully align with a canonical miRNA-sponge/ceRNA model. Prior work has shown that some circRNAs (e.g., CDR1as) may stabilize bound miRNAs rather than simply sequestering them, leading to non-canonical regulatory outcomes (Piwecka et al., 2017). Accordingly, we speculate that circMPP6 may help maintain hsa-miR-188-3p and hsa-miR-1305 levels/activity, thereby supporting sustained repression of SLC7A11. This hypothesis requires further validation by dedicated mechanistic assays (e.g., AGO2 RIP/CLIP, binding-site mutagenesis, and miRNA rescue experiments).

Beyond NSCLC, circMPP6 has been implicated in other malignancies, acting via miRNA sequestration (prostate cancer) (Pan et al., 2022) or scaffolding of protein complexes to modulate mRNA decay (colorectal cancer) (Chen et al., 2024). These mechanistic precedents underscore the versatility of circRNAs and suggest that circMPP6 may deploy cell-contextual mechanisms, with SLC7A11 regulation representing one downstream axis in lung adenocarcinoma.

This study has limitations. First, most mechanistic and phenotypic data were generated in A549 and H1975 cells. Given interline heterogeneity (e.g., KRAS/TP53 status, basal SLC7A11 expression), validation across additional NSCLC models (such as H1299, H1975, HCC827) is warranted. Second, the molecular mechanism by which circMPP6 represses SLC7A11 remains unresolved. Future work should prioritize: (i) testing a ceRNA hypothesis via miRNA profiling and AGO2-RIP, dual-luciferase reporters, and rescue by miRNA mimics/inhibitors; (ii) exploring RBP-dependent mechanisms through RNA pulldown-mass spectrometry and CLIP-seq; (iii) assessing transcriptional effects using promoter/enhancer assays and chromatin engagement of relevant TFs. Third, the clinical cohort was small; larger, stage-stratified cohorts with matched multi-omics will be important to confirm the disrupted circ-linear correlation and its prognostic value. Finally, while our data support SLC7A11 as a downstream effector, causal mediation should be tested with epistasis assays. In addition, we also explored the prognostic relevance of circMPP6 by deriving a gene-set model from circMPP6-regulated mRNAs, as large-scale circRNA datasets are still limited whereas mRNA data are widely available. This indirect, exploratory approach did not fully reproduce the prognostic association expected from our functional findings, highlighting the complexity of the circMPP6 regulatory network and the limitations of inferring circRNA activity solely from target gene expression.

In sum, our results reveal a functional divergence between circMPP6 and its parental gene MPP6 in NSCLC. circMPP6 counteracts MPP6-driven pro-proliferative and redox programs, in part via suppression of SLC7A11 and GSH biosynthesis, highlighting an antagonistic circ-linear axis with potential therapeutic implications. Targeting MPP6-high tumors by restoring circMPP6 function or inhibiting SLC7A11 may represent testable strategies, pending mechanistic validation and *in vivo* efficacy studies.

## Conclusion

circMPP6 and its parental gene MPP6 converge on hypoxia-linked pathways yet exert distinct—and in part opposing—effects on NSCLC growth and metabolism. In the setting of MPP6 overexpression, circMPP6 functions as a counter-regulatory, tumor-suppressive factor, potentially through downregulation of SLC7A11 and attenuation of GSH-dependent redox buffering.

## Data Availability

The original contributions presented in the study are included in the article/Supplementary Material, further inquiries can be directed to the corresponding authors.

## Supplementary material

SUPPLEMENTARY FIGURE S1 MPP6 expression in non-small cell lung cancer (NSCLC) from TCGA and CPTAC datasets. **(A, B)** Boxplots of MPP6 mRNA expression in lung adenocarcinoma (LUAD) and lung squamous cell carcinoma (LUSC) versus normal lung tissues from TCGA/GTEx as analyzed by GEPIA. In **(A)**, a threshold of |log2FC| > 0.5 and P < 0.01 was used; in **(B)**, a more stringent cutoff of |log2FC| > 1 and P < 0.01 was applied. **(C,D)** The protein expression levels (Z-score) of MPP6 in CPTAC cohorts were analyzed in both LUSC and LUAD.

SUPPLEMENTARY FIGURE S2 Overexpression of MPP6 and circMPP6: effects on cell proliferation and downstream SLC7A11 expression. **(A)** qRT-PCR validation of the overexpression of MPP6 and circMPP6 in H1975 cells. **(B-D)** CCK-8 assay results showing the effects of MPP6 **(B)** and circMPP6 **(C)** overexpression, as well as the co-overexpression of MPP6 and circMPP6 **(D)**, on the proliferation of H1975 cells. **(E,F)** Western blot analysis of SLC7A11 expression in H1975 cells following overexpression of circMPP6 **(E)** and MPP6 **(F**).

SUPPLEMENTARY FIGURE S3 circMPP6 and MPP6 exert opposing effects on hypoxia-related genes in both A549 and H1975 cell lines. **(A)** In A549 cells, overexpression of circMPP6 under CoCl_2_ treatment altered the expression of hypoxia-related genes DDIT4, STC2, ANGPTL4, and NDRG1. **(B)** Similarly, in H1975 cells, circMPP6 overexpression under CoCl_2_ treatment modulated the expression of DDIT4, STC2, ANGPTL4, and NDRG1. **(C)** In A549 cells, Overexpression of MPP6 under CoCl_2_ treatment also affected the expression of DDIT4, STC2, ANGPTL4, and NDRG1. **(D)** Likewise, in H1975 cells, MPP6 overexpression under CoCl_2_ treatment influenced the expression of the same gene set (DDIT4, STC2, ANGPTL4, and NDRG1).

SUPPLEMENTARY FIGURE S4 Pathway enrichment of chromosome-specific DEGs regulated by circMPP6 Integrated KEGG (green) and Hallmark (blue) enrichment analyses for chromosome-specific DEG sets under circMPP6 overexpression. Only chromosomes with significant enrichment are displayed; bubble size indicates gene ratio and color indicates FDR.

SUPPLEMENTARY FIGURE S5 Pathway enrichment of chromosome-specific DEGs regulated by MPP6 Integrated KEGG (green) and Hallmark (blue) enrichment analyses for chromosome-specific DEG sets under MPP6 overexpression. Only chromosomes with significant enrichment are displayed.

SUPPLEMENTARY FIGURE S6 Impact of MPP6 and circMPP6 on cell proliferation under hypoxia. **(A)** CCK-8 assay of A549 cell proliferation following MPP6 overexpression under CoCl_2_-induced hypoxia. **(B)** CCK-8 assay of A549 cell proliferation following circMPP6 overexpression under CoCl_2_-induced hypoxia. **(C)** CCK-8 assay of H1975 cell proliferation following MPP6 overexpression under CoCl_2_-induced hypoxia. **(D)** CCK-8 assay of H1975 cell proliferation following circMPP6 overexpression under CoCl_2_-induced hypoxia.

SUPPLEMENTARY FIGURE S7 Prognostic modeling based on circMPP6-regulated genes in LUAD. **(A)** Construction of a prognostic model in the TCGA-LUAD training cohort; schematic and model workflow. **(B)** Forest plot showing hazard ratios and 95% CIs for circMPP6-regulated genes included in the multivariable Cox model. **(C,D)** External validation in LUAD cohorts GSE72094 **(C)** and GSE68465 **(D)**; Kaplan-Meier survival curves comparing risk groups, with log-rank P values. **(E,F)** Association of MPP6 expression with disease-free survival **(E)** and overall survival **(F)** in TCGA-LUAD; KM curves with log-rank P values.

SUPPLEMENTARY FIGURE S8 Prediction of candidate miRNAs and RBPs associated with circMPP6. **(** **A-C)** Based on the miRanda and TargetScan algorithms, miRNAs harboring putative binding sites on circMPP6 were predicted using the CircAtlas **(A)** and CircInteractome **(B)** databases. **(C)** Overlapping miRNAs from the intersection analysis of both datasets. **(D)** Analysis of RBPs with potential interactions to circMPP6 was performed using the CircAtlas database.

SUPPLEMENTARY FIGURE S9 circMPP6 may regulate SLC7A11 expression by binding to miR-188-3p or miR-1305. **(A)** Prediction of potential binding sites between the SLC7A11 3'UTR and circMPP6. **(B)** RNA pulldown assays confirmed the miRNAs that bind to circMPP6.
