# Supplementary figures and images for: Divergent roles of circMPP6 and its parental gene MPP6 in non-small cell lung cancer
Source: Front Cell Dev Biol. 2026 Feb 3;14:1722916. doi: 10.3389/fcell.2026.1722916 (PMC12960637; doi:10.3389/fcell.2026.1722916)

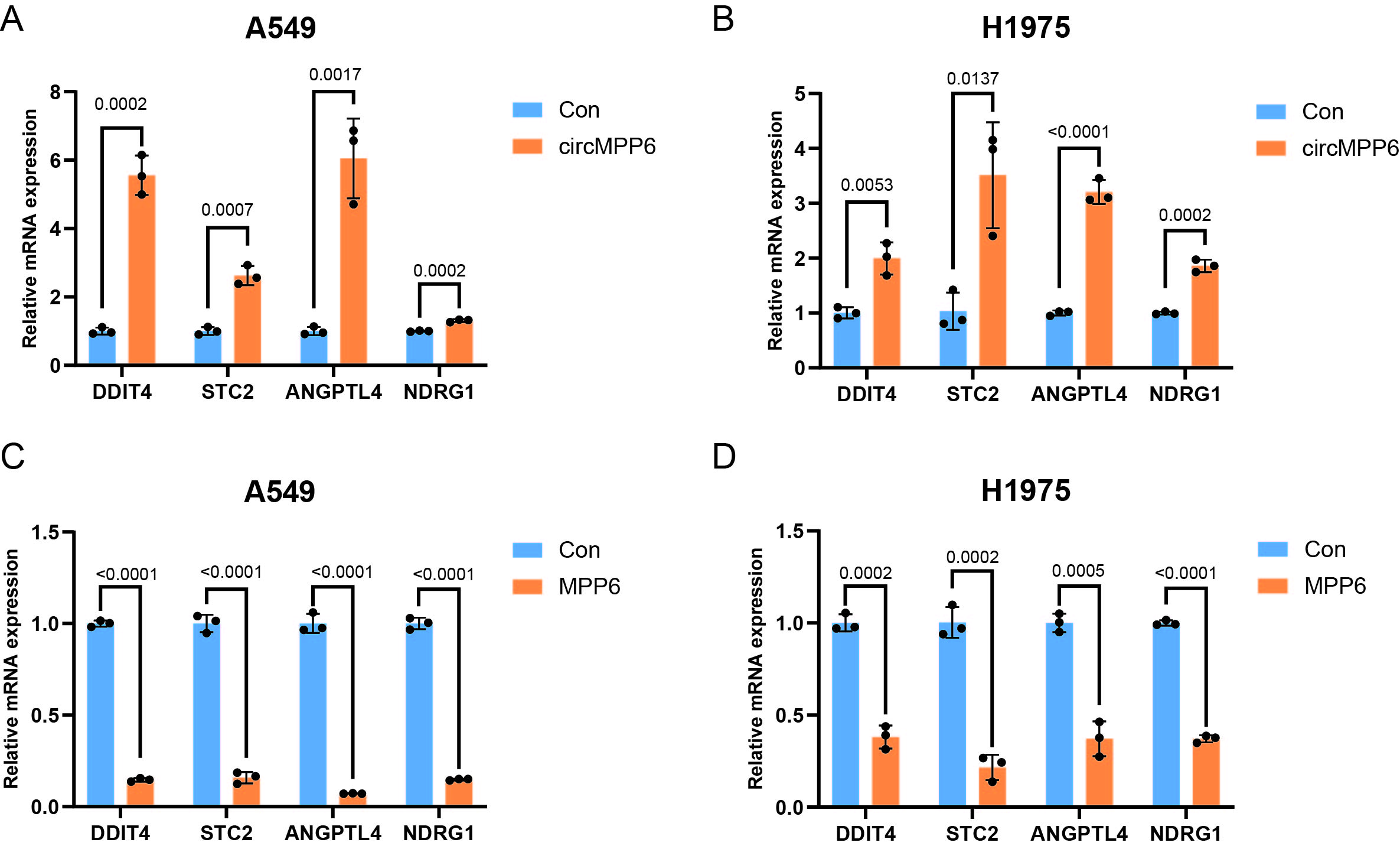

Supplement: Supplementary file 1 [file Image3.jpeg]

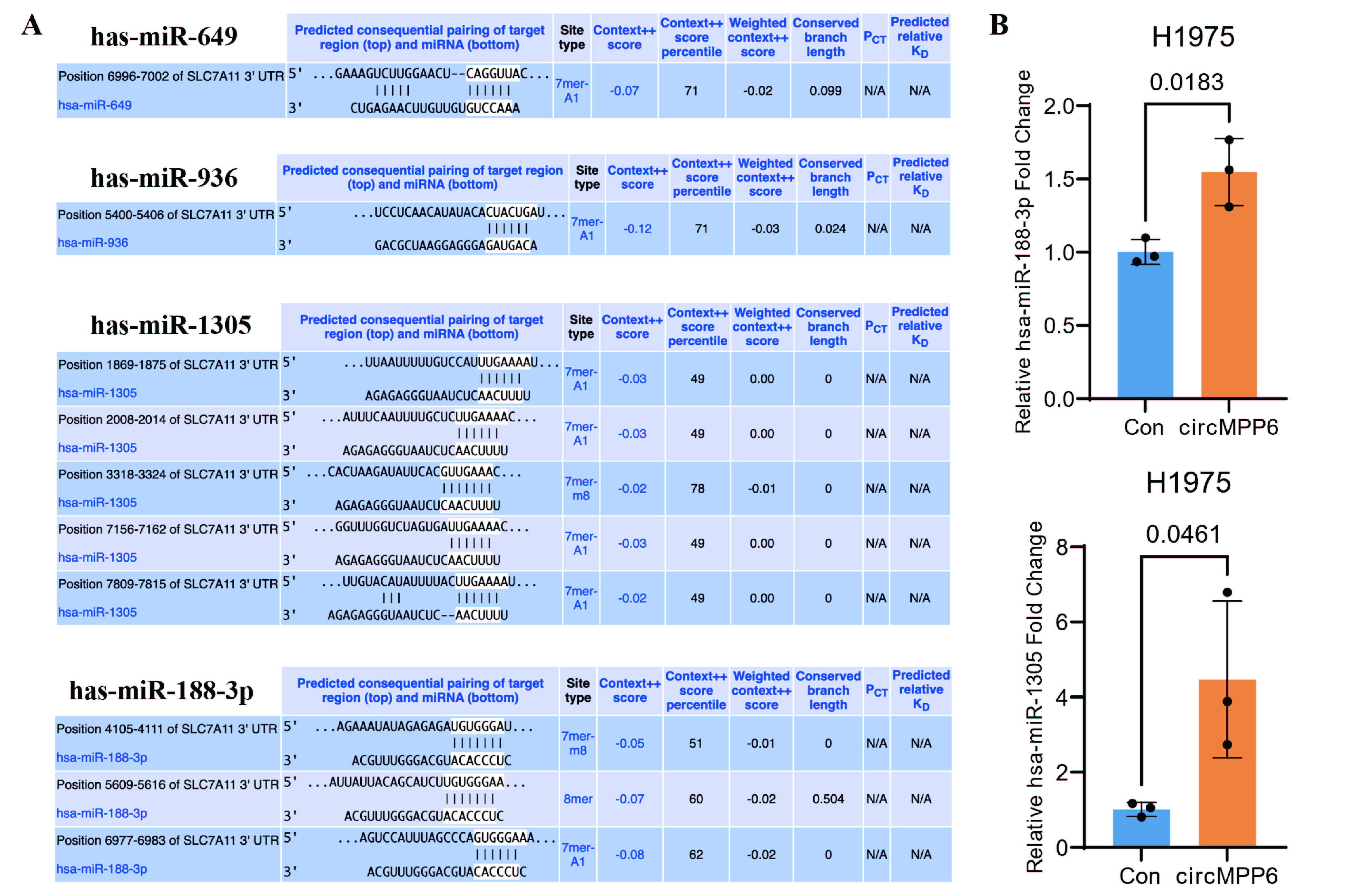

Supplement: Supplementary file 2 [file Image9.jpeg]

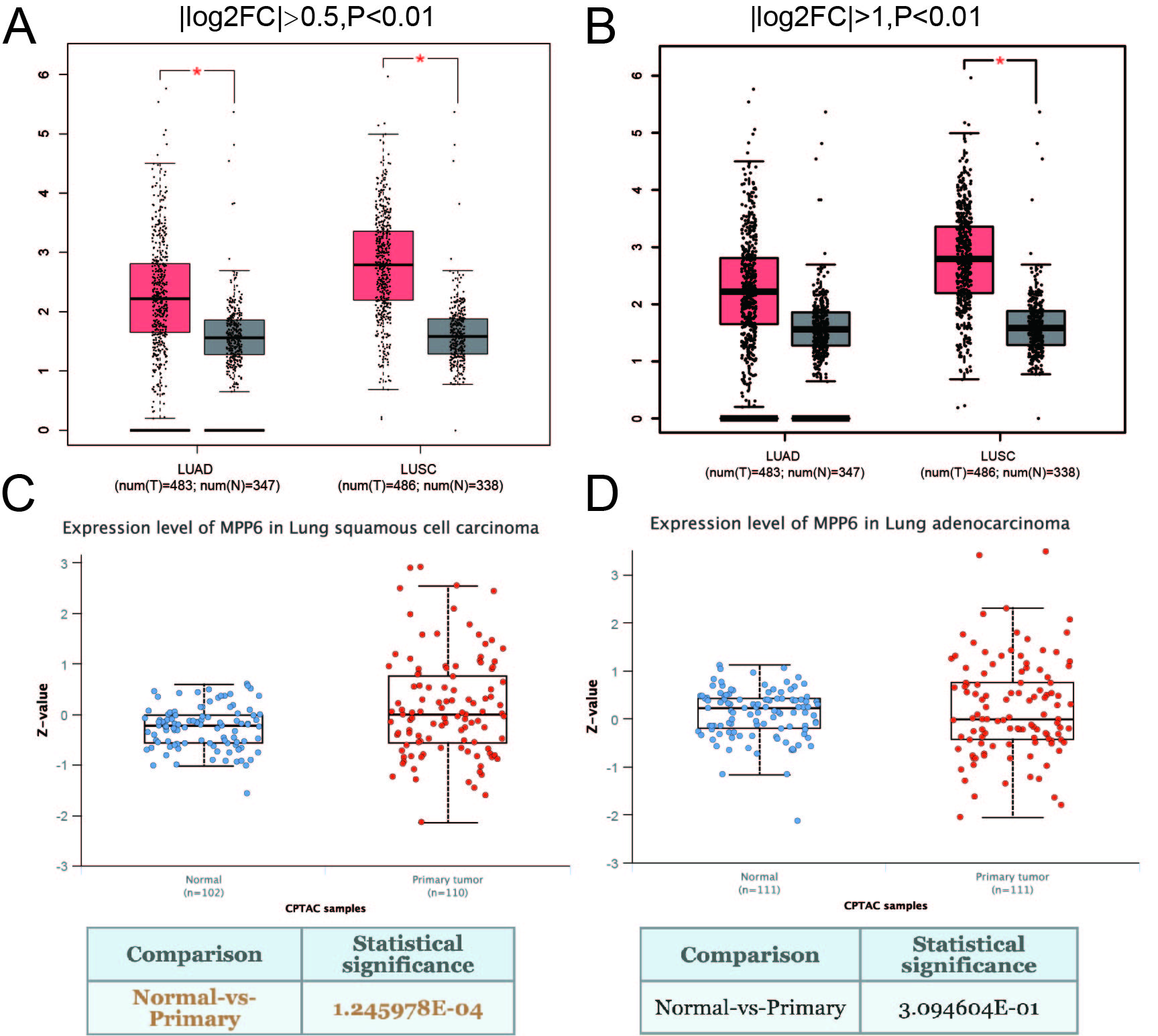

Supplement: Supplementary file 3 [file Image1.jpeg]

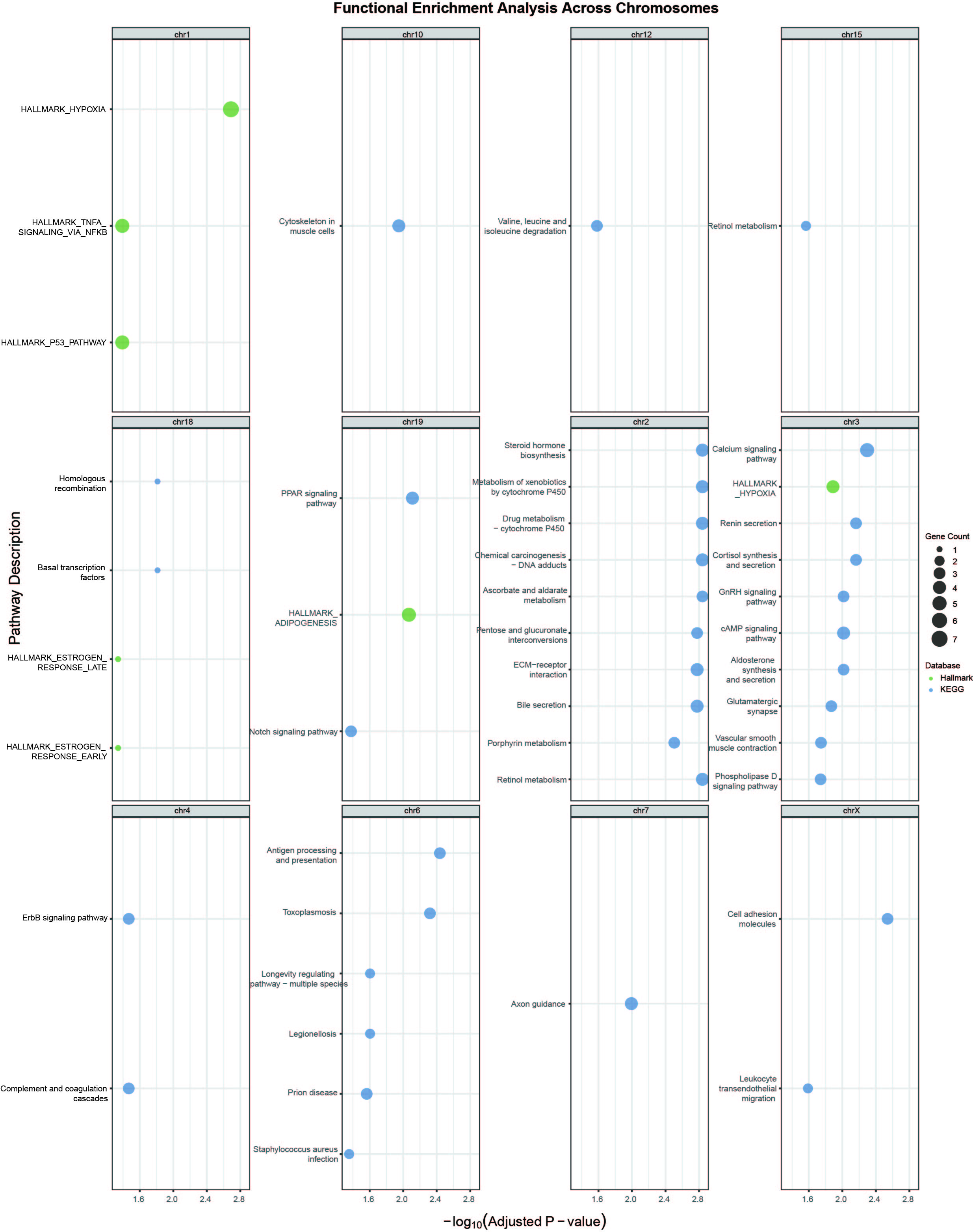

Supplement: Supplementary file 4 [file Image4.jpeg]

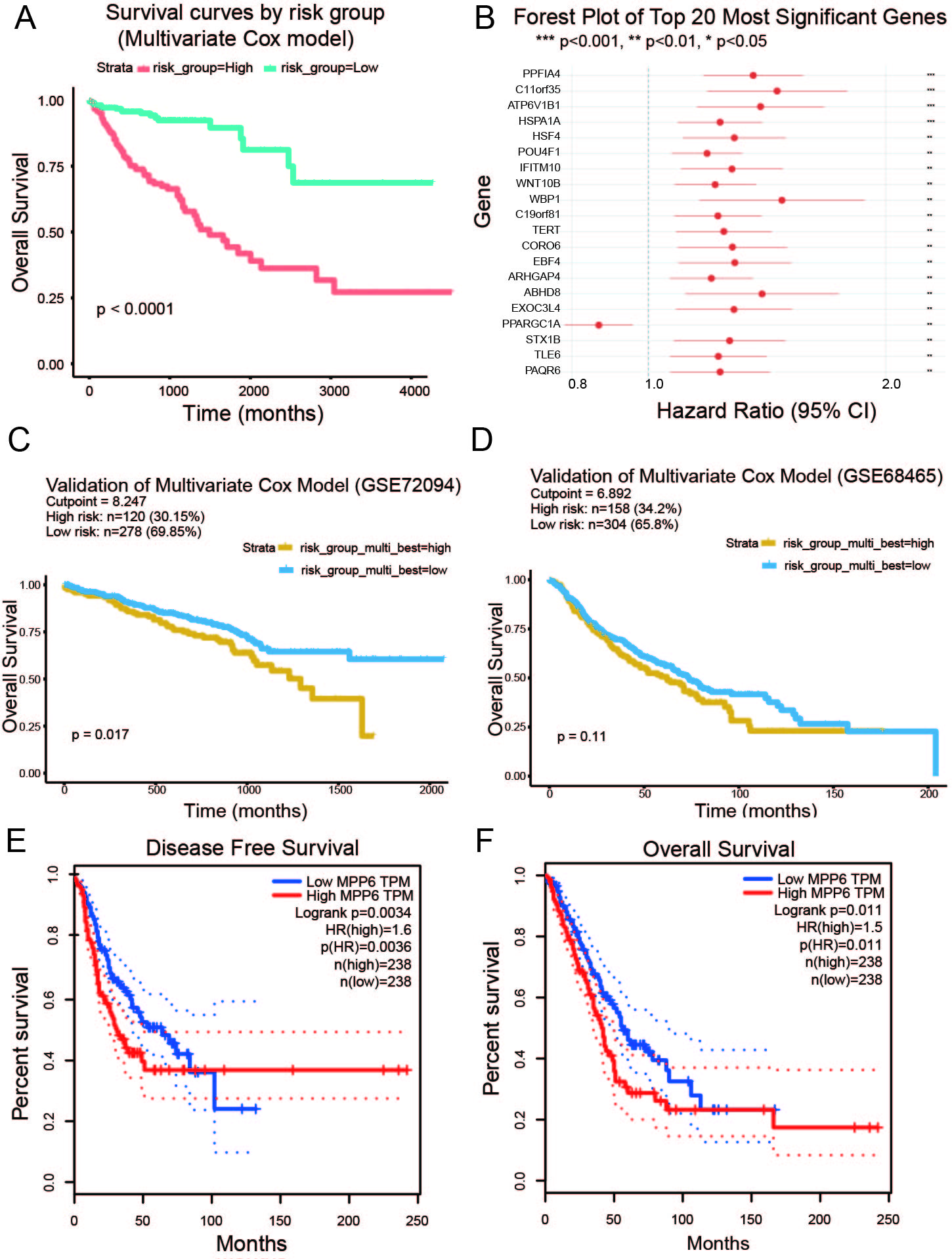

Supplement: Supplementary file 5 [file Image7.jpeg]

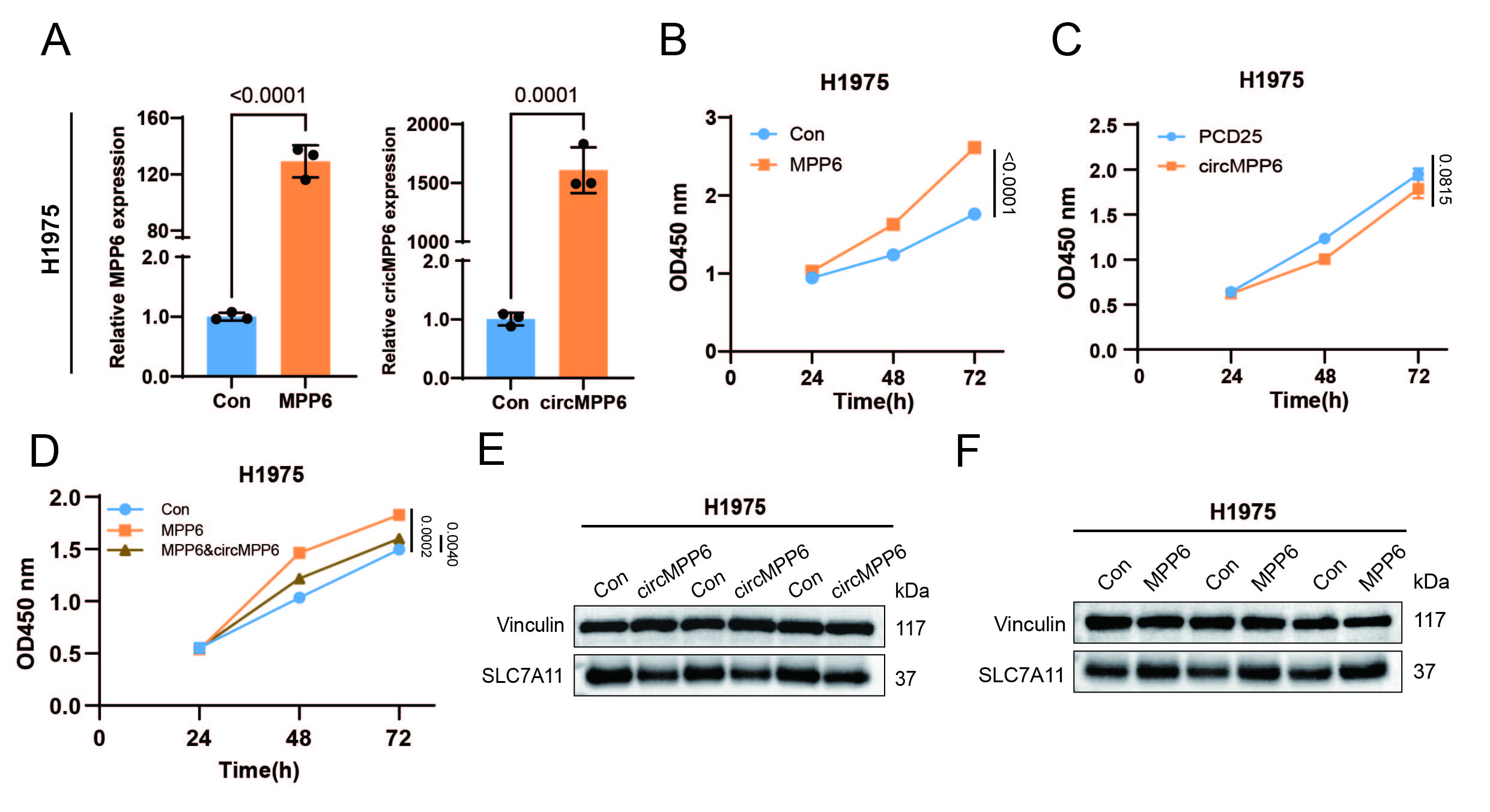

Supplement: Supplementary file 6 [file Image2.jpeg]

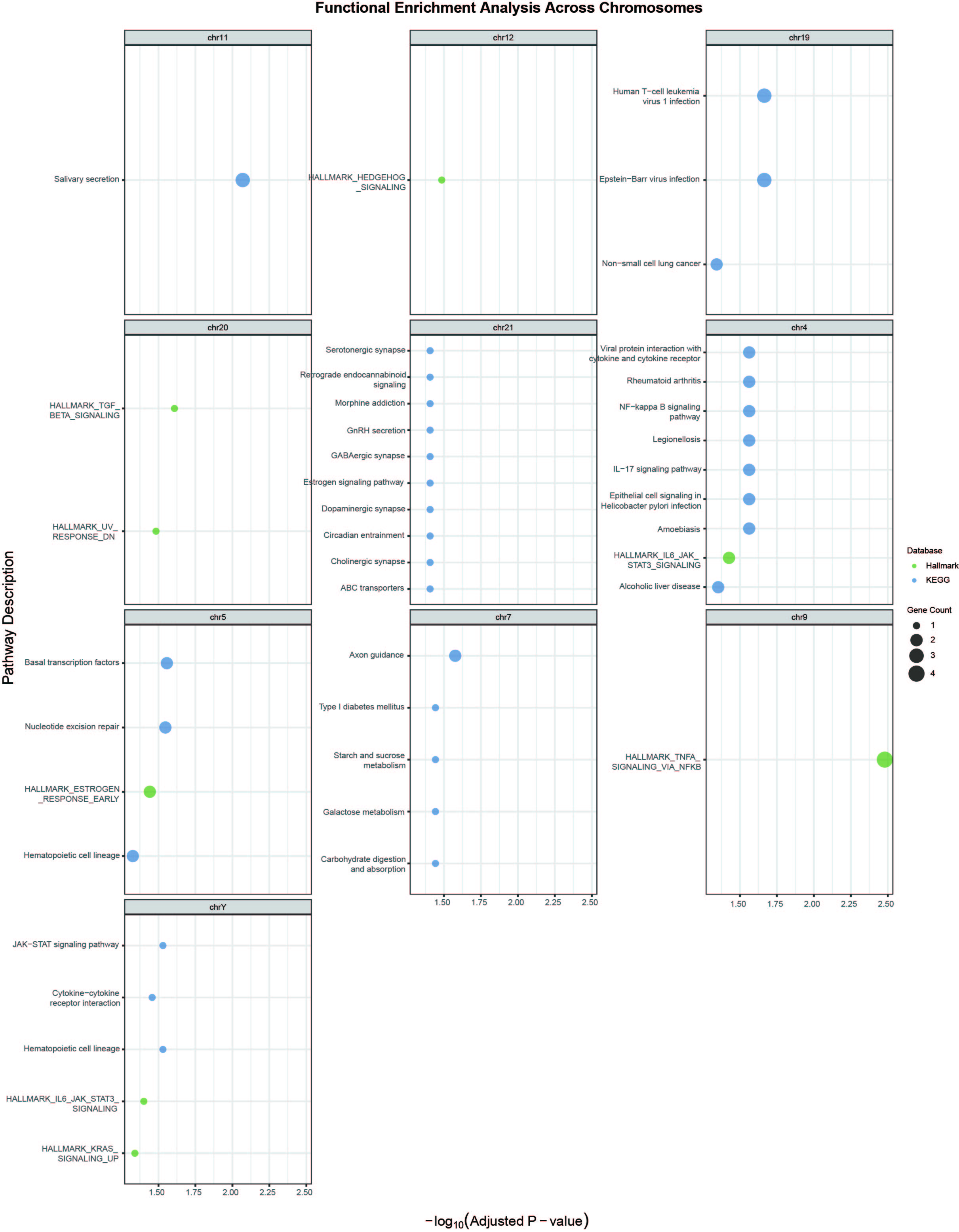

Supplement: Supplementary file 7 [file Image5.jpeg]

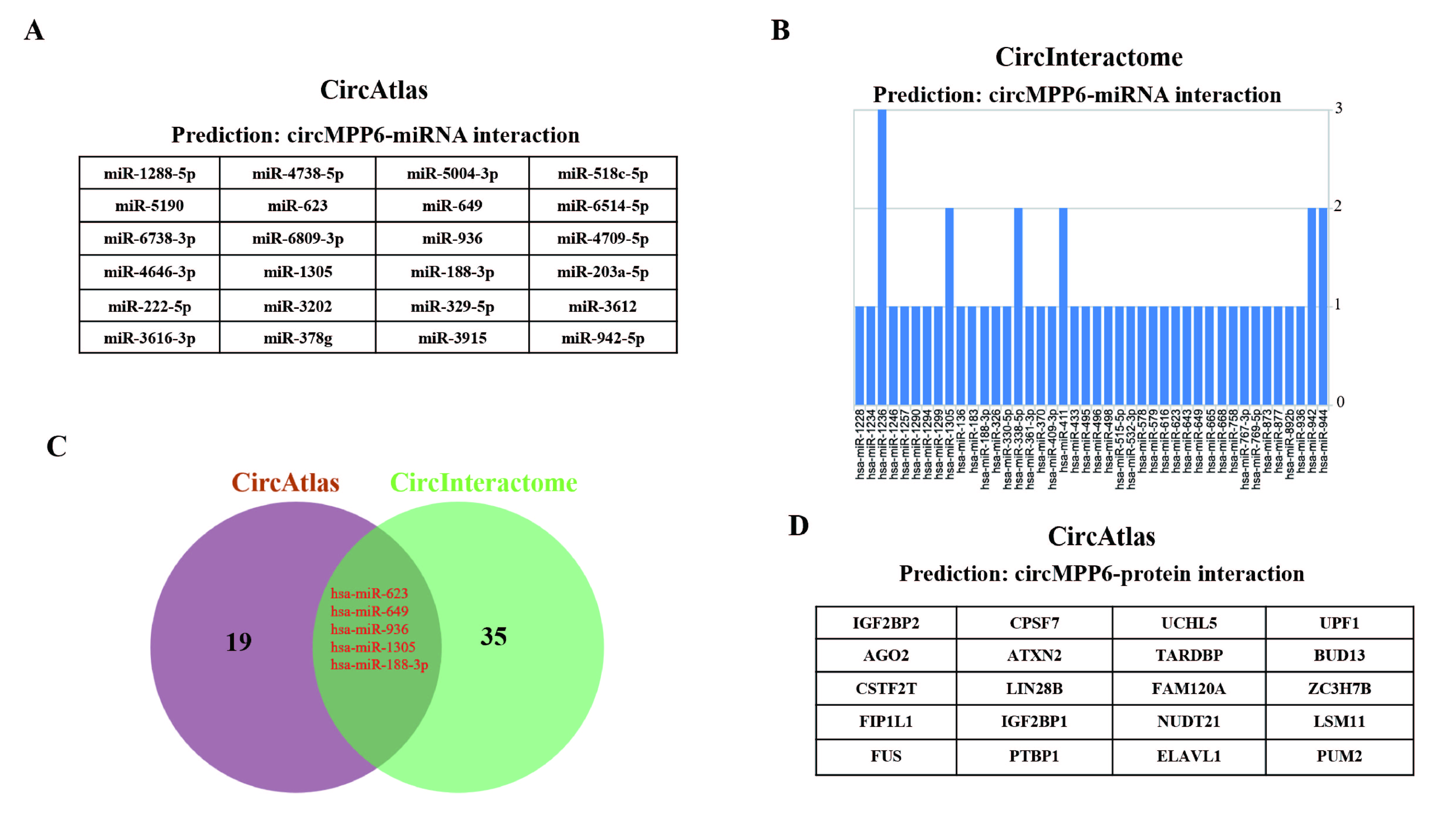

Supplement: Supplementary file 8 [file Image8.jpeg]

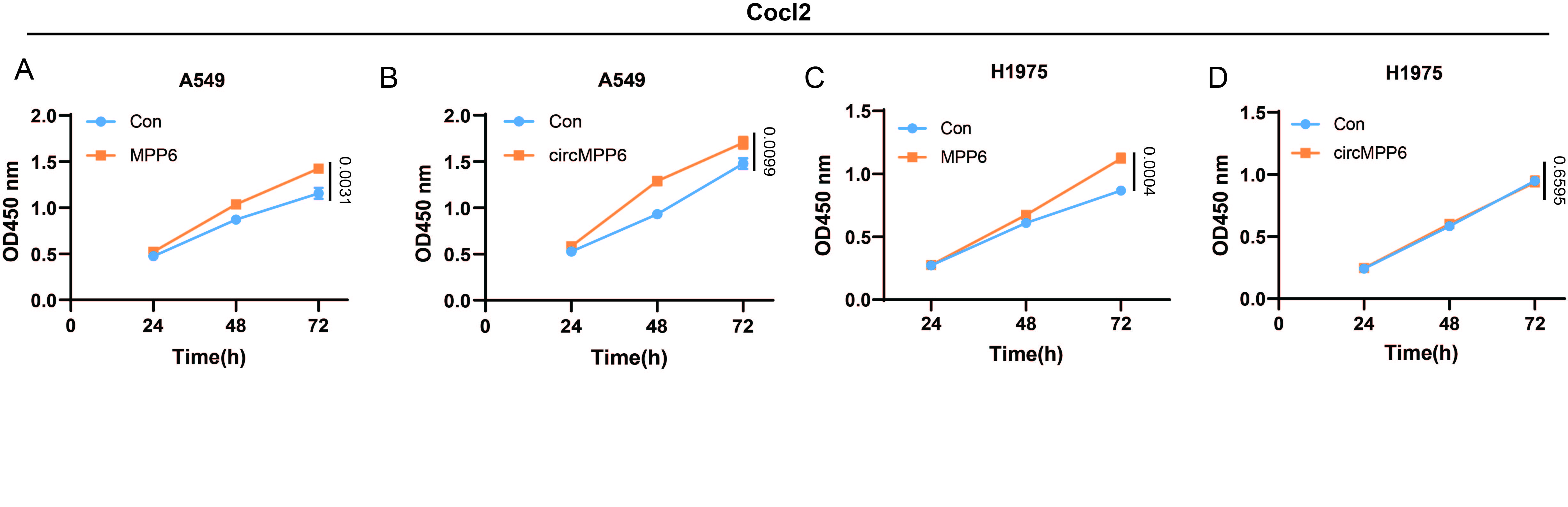

Supplement: Supplementary file 9 [file Image6.jpeg]
